# Epidemiology and Clinical Outcomes of Inflammatory Bowel Disease: A Hospital-Based Study in Central Taiwan

**DOI:** 10.1155/2019/4175923

**Published:** 2019-06-13

**Authors:** Jen-Wei Chou, Hsiang-Chun Lai, Chia-Hsi Chang, Ken-Sheng Cheng, Chun-Lung Feng, Tsung-Wei Chen

**Affiliations:** ^1^School of Medicine, China Medical University, Taichung, Taiwan; ^2^Division of Gastroenterology and Hepatology, Department of Internal Medicine, China Medical University Hospital, Taichung, Taiwan; ^3^Taiwan Society of Inflammatory Bowel Disease, Taichung, Taiwan; ^4^Taiwan Association for the Study of Small Intestinal Diseases, Taoyuan, Taiwan; ^5^Department of Chinese Medicine, China Medical University Hospital, Taichung, Taiwan; ^6^Department of Pathology, China Medical University Hospital, Taichung, Taiwan

## Abstract

The incidence and prevalence of inflammatory bowel disease (IBD) are low but increasing in Taiwan. We aimed to investigate the epidemiology and clinical outcomes of IBD in central Taiwan. We retrospectively analyzed patients with IBD diagnosed at our hospital between January 2000 and September 2018. The diagnostic criteria were based on endoscopic and pathologic findings. Clinical characteristics, treatment regimens, and treatment outcomes were analyzed. A total of 190 patients with IBD were enrolled (80 with Crohn's disease (CD) and 110 with ulcerative colitis (UC)). The mean age at diagnosis was 38.4 years (CD: 36 years, UC: 40 years). Male patients accounted for the majority of patients (71.1%). The male-to-female ratio was 3 : 1 for CD and 2.1 : 1 for UC. Current and ever smokers accounted for 30.5% of all patients. Only 4.2% of patients had a family history of IBD. Extraintestinal manifestations (EIMs) were reported in 7.9%, and colorectal cancers (CRCs) were reported in 2.1% of all patients. In patients with CD, the ileal type was the most common disease phenotype (57.5%), and the stricturing type was the most common disease behavior (60.0%). In patients with UC, left-sided colitis was the predominant disease extent (42.7%). The seroprevalence of hepatitis B virus (HBV) was 13.3%. The incidence of perinuclear anti-neutrophil cytoplasmic antibody (p-ANCA) in patients with UC was 22%. 5-Aminosalicylic acids were the preferred treatment for UC, whereas corticosteroids, immunomodulators, and biologic agents were preferred for CD. In patients with CD, the bowel resection rate was 38.8%, and the incidence of hip avascular necrosis was 3.8%. In Taiwan, patients with IBD showed a male predominance, lack of familial clustering, a higher prevalence of HBV infection, and a lower prevalence of p-ANCA, EIMs, and CRC. Moreover, a higher incidence of the ileal type with poor outcomes of CD and left-sided predominance in UC were found.

## 1. Introduction

Inflammatory bowel disease (IBD), which includes Crohn's disease (CD) and ulcerative colitis (UC), is a chronic relapsing and remitting disease. Although there have been numerous epidemiologic studies on IBD, the etiology of this disease remains unclear. UC mainly affects the colon and rectum, whereas CD can affect the entire gastrointestinal (GI) tract. IBD was believed to be a Western disease, as it is most prevalent in North America, Europe, and Oceania. The prevalence of IBD in the Western world is about 50–200 per 100,000 persons for CD and 120–200 per 100,000 persons for UC [1]. However, the incidence and prevalence rates of IBD have shown an increasing trend in Asia and Africa [2]. Therefore, IBD has become a global disease in the 21st century with a large socioeconomic burden [2, 3].

The incidence and prevalence of IBD are low in Taiwan compared with Western countries, but they have shown an increasing trend in the recent 2 decades [3, 4]. This trend might be attributed to the Westernized lifestyle [4]. In East Asia, there are unique regional environmental factors, such as a higher prevalence of hepatitis B virus (HBV) and tuberculosis infection, which cause some differences in the characteristics of patients with IBD. Moreover, it has been reported that UC has milder severity in Asia [5]. In the literature on IBD in Taiwan, most of the epidemiologic studies are population-based studies that used data from the Taiwan National Health Insurance Database, which tend to underestimate the true prevalence and incidence. However, hospital-based studies on IBD are scarce. Population-based studies may have some errors because of incorrect coding. Moreover, such studies may lack details of clinical characteristics and treatment programs. In our present study, we aimed to investigate the clinical characteristics and treatments of patients with IBD through a hospital-based study in central Taiwan.

## 2. Materials and Methods

### 2.1. Study Population

We searched the database of chart records of China Medical University Hospital (CMUH), a tertiary medical center in central Taiwan, from January 2000 to September 2018. We also searched the International Classification of Disease (2001 version) for disease coding, according to which UC is coded as 556.XX and CD as 555.XX in our chart records. We enrolled patients with a regular follow-up for at least 3 months after the original diagnosis of IBD. All data were completely reviewed by Dr. Chou, a gastroenterologist at CMUH. The diagnostic criteria for IBD were based on a combination of clinical presentations, endoscopic features, and pathologic findings (infectious and malignant etiologies were excluded). The follow-up duration of our patients was from the time of diagnosis or the first hospital visit for treatment to the last record in our chart.

We collected clinical data including sex, age at diagnosis, alcohol consumption/smoking habits, family history, disease phenotype, and behavior at diagnosis according to the Montreal classification, extraintestinal manifestations (EIMs), presence of fistula, colorectal cancers (CRCs), avascular necrosis (AVN) of the hips, hepatitis B virus (HBV)/hepatitis C virus (HCV) infection status, and anti-neutrophil cytoplasmic antibody (ANCA) status. The clinical course of the patients was recorded and analyzed. We also collected and analyzed the treatment regimens and bowel resection rates in our patients with IBD.

### 2.2. Statistical Methods

For statistical methods, we used Pearson's chi-square test for categorical variables and Student's *t*-test for continuous variables.

### 2.3. Ethical Considerations

This study was approved by the institutional review board of the research ethics committee of CMUH in central Taiwan (CMUH107-REC1-139).

## 3. Results

A total of 190 patients with IBD were enrolled in our present study. The clinical characteristics of all patients are shown in Table 1. There were 80 patients with CD and 110 patients with UC. The mean age at diagnosis of all patients was 38.4 ± 15.9 years (range: 9–85 years). Although the mean age at diagnosis of patients with CD was lower than that of patients with UC, there was no statistical significance between these 2 groups (CD: 36 years vs. UC: 40 years, *p* = 0.080). With respect to the sex distribution, male patients accounted for the majority (71.1%) of all patients. The male-to-female ratio was 3 : 1 in the CD group and 2.1 : 1 in the UC group. The proportion of male patients showed no significant difference between the CD and UC groups (CD: 75.0% vs. UC: 68.2%, *p* = 0.306). With respect to the smoking status, the proportion of current smokers, ever smokers, and nonsmokers was 13.7%, 16.8%, and 69.5%, respectively. There was a significant difference in the proportion of patients with a smoking habit between the CD and UC groups (*p* = 0.002). Concerning alcohol consumption, the incidence of current drinking, social drinking, ever drinking, and nondrinking was 1.6%, 11.1%, 5.3%, and 82.1%, respectively. There was no statistical difference between the CD and UC groups in the habit of alcohol consumption (*p* = 0.194). In the investigation of family history of IBD in all patients, we found only 4.2% with a family history, with no significant difference between the CD and UC groups (CD: 2.5% vs. UC: 5.5%, *p* = 0.317). In the analysis of EIMs, the incidence of EIM was 7.9% in all patients with IBD. There was no significant difference in the incidence of EIM between the CD and UC groups (CD: 8.8% vs. UC: 7.3%, *p* = 0.709). The incidence of CRC in all patients was 2.1%. There was no significant difference between patients with CD and those with UC (CD: 1.2% vs. UC: 2.7%, *p* = 0.484). There were some missing data in laboratory tests, including HBV/HCV infection status and serum ANCAs. We analyzed these variables by excluding missing data. The incidence of HBV infection was 13.3% in 158 patients with IBD with available data. However, there was no significant difference between the CD and UC groups (CD: 11.3% vs. UC: 15.4%, *p* = 0.444). The incidence of perinuclear ANCA (p-ANCA) was 22% and that of cytoplasmic ANCA (c-ANCA) was 1.6% in 63 patients with UC with available data.

The Montreal classifications of all patients with IBD in our study are summarized in Table 2. With respect to the disease phenotype of CD, we found 57.5%, 7.5%, 33.8%, and 1.3% of cases to be located in L1 (ileum), L2 (colon), L3 (ileo-colon), and L4 (upper GI tract) at diagnosis, respectively. Concerning the disease behavior of CD, B1 (nonstricturing and nonpenetrating type), B2 (stricturing type), and B3 (penetrating type) were observed 18.8%, 60.0%, and 21.3%, respectively. The ileal type and stricturing type accounted for the majority of our CD cases. In the disease extension of UC, we found that the incidence of E1, E2, and E3 at diagnosis was 18.2%, 42.7%, and 39.1%, respectively. Left-sided UC accounted for the majority of UC cases in our study. Of the patients with CD, 17 (21.3%) had perianal involvement and 22 (27.5%) were complicated with fistula formation.

The treatment regimens, bowel resection rates, and hip AVN incidence in our patients with IBD are summarized in Table 3. The treatment regimens for IBD in our present study included 5-aminosalicylic acids (5-ASAs), corticosteroids, immunomodulators, and biologic agents. Our data showed that 5-ASAs were more preferred in patients with UC than in those with CD. There was a statistical difference between the UC and CD groups (UC: 99.1% vs. CD: 93.8%, *p* = 0.038). Moreover, the uses of corticosteroids (CD: 80.0% vs. UC: 61.8%, *p* = 0.007), immunomodulators (CD: 63.8% vs. UC: 27.3%, *p* < 0.001), and biologic agents (CD: 85.0% vs. UC: 42.7%, *p* < 0.001) all showed a statistically significant difference between patients with CD and those with UC. In our study, the bowel resection rate in all patients with IBD was 18.4%. Patients with CD showed a significantly higher bowel resection rate than those with UC (CD: 38.8% vs. UC: 3.6%, *p* < 0.001). The prevalence of hip AVN in all patients was 3.2%. Although patients with CD showed a trend of having more AVN complications than patients with UC, there was no significant difference between the groups (CD: 3.8% vs. UC: 2.7%, *p* = 0.484).

## 4. Discussion

IBD used to be known as a Western disease because of its higher prevalence in North America, Europe, and Oceania [3]. Recent reports demonstrated that the incidence and prevalence rates of IBD in Asia have been increasing [6]. Wei et al. reported an epidemiologic study of IBD from 1998 to 2008 in Taiwan. They found that the prevalence of CD increased from 0.19/100,000 to 1.78/100,000 and that of UC increased from 0.61/100,000 to 7.62/100,000 [7]. These results may be attributed to urbanization, lifestyle changes, diet, and advanced diagnostic methods such as enteroscopy [8]. Thus, IBD has become an important issue in Asian people.

With respect to the prevalence of IBD types, UC seemed to be more prevalent than CD (202/100,000 vs. 146/100,000) [9]. In our study, we found comparable results for the UC and CD ratios. Several cohort studies have indicated a female predominance in CD and a male predominance in UC [1]. The sex distribution of IBD has been consistently significantly different worldwide. IBD is a male-predominant disease in Asia, according to previous studies. In our present study, we also observed a male predominance (75.0% in CD and 68.2% in UC). These ratios were higher than those reported in previous Asia-Pacific studies, in which male patients constituted 59.6% of CD cases and 57.7% of UC cases [8]. However, a Korean study reported that male patients constituted 73% of CD cases, which is comparable to our data [6]. With respect to the age at diagnosis of IBD, the peak age at onset for CD was between 20 and 30 years and that for UC was between 30 and 40 years [10]. Our patients tended to be diagnosed at an older age with CD but at a younger age with UC (CD: 36.0 years, UC: 40.1 years) compared with the patients in another study (CD: 33 years, UC: 43 years) [8]. A consistent trend was observed that patients with CD had a younger age at diagnosis than did patients with UC. Nevertheless, there was no significant difference between the ages of patients with CD and those with UC. The reason may be the small number of our patients. Smoking is one of the known environmental protective factors for UC, but it is a risk factor for CD [11]. The higher prevalence rate of cigarette smoking also had an association with lower income levels in America, Europe, and Asia [12]. Birrenbach and Bocker reported a wide range of prevalence rates of cigarette use in patients with IBD (CD: 39–72%, UC: 11–23%) and found that nonsmokers were more common among patients with UC than among those with CD [13]. Our present study found that among patients with CD, 23.8% were current smokers and 13.8% were ever smokers, and these percentages were higher than those reported in another Asia-Pacific study (11.6% current smokers and 11.3% ever smokers) [8]. Men have a greater tendency to smoke than women in Asia [14]. This high smoking rate in patients with CD might have partially contributed to the higher proportion of male patients in our study; however, more studies are needed to prove this assumption. In our study, there were 6.4% current smokers and 19.1% ever smokers among patients with UC, and these percentages were comparable to those reported in a previous study [8]. Some Asian cohort studies demonstrated that smoking was a protective factor against UC [15]. In the English literature, we found fewer data on alcohol consumption than on tobacco usage among patients with IBD. The incidence of alcohol consumption in our results was lower than that in previous studies (39.5% ever drinking and 46.9% binge drinking) [16, 17]. Additionally, evidence showing that alcohol consumption exacerbates IBD remains equivocal. Some studies reported that alcohol is a sulfur and sulfate producer, thus increasing the concentration of fecal hydrogen sulfide (an agent toxic to colonocytes) [18–20]. Patients with IBD in Asia tend to have a lower positive family history rate (0.0–3.4%) than those in Western countries (10–25%) [2, 21, 22]. The rate of positive family history of IBD (4.2%) in our study was consistent with that in other Asian reports. However, recent Asian studies demonstrated an increased positive family history rate of IBD patients, which might be attributable to a higher recognition of IBD [23].

With respect to the disease phenotype of CD according to the Montreal classification, equal proportions of phenotypes were reported in patients with CD, whereas some studies reported that the colonic type was predominant in Western people. Prideaux et al. demonstrated that the ileo-colonic type was the most common type of CD in Asia (about 30–50%), followed by the ileal and colonic types [2]. Ng et al. reported more equal distributions of the ileal, colonic, and ileo-colonic types (31.7%, 24.4%, and 43.9%, respectively) and that the incidence of the upper GI type was 6%, consistent with previously reported hospital-based data in Taiwan. In our study, we identified that the most common phenotype was the ileal type, followed by the ileo-colonic, colonic, and upper GI types. Concerning the disease behavior of CD, the nonstricturing and nonpenetrating type was the most common type in Western reports (62–81%) compared with Asian reports (40–69%) [2, 8, 24]. In our study, we found that the most dominant disease behavior was the stricturing type, followed by the penetrating type and then the nonstricturing and nonpenetrating type. Our results showed that patients with CD in Taiwan had higher ratios of the stricturing and penetrating types at diagnosis, with poor prognostic outcomes. The first reason may be the delay in diagnosis owing to the lack of awareness of CD. The second reason is that ileal-type CD is difficult to diagnose because of the need for small-bowel endoscopy, which is a challenging procedure for most endoscopists in Taiwan. In the literature, the incidence of perianal involvement ranges from 15.6% to 36.7% in patients with CD [2, 8]. We reported an incidence of 21.3%, which was comparable to that in a previous report in Taiwan [24]. Schwartz et al. demonstrated that 20–40% of patients with CD developed a fistula (mostly perianal fistula followed by enteroenteric fistula) and the cumulative risk of any fistula formation was 50% after 20 years [25]. In our study, 27.5% of all patients had a fistula (mainly anal fistula formation), comparable to the report by Schwartz et al. With respect to the disease extent of UC, large variations have been reported worldwide. Western reports showed proctitis in 30–60%, left-sided colitis in 16–40%, and extensive colitis in 18–35% of patients with UC. However, Asia-Pacific reports demonstrated equal distributions among these 3 types (32.6%, 34.9%, and 36.2%, respectively) [2, 8]. In the present study, the most common type was left-sided UC, followed by extensive colitis and proctitis, which was comparable to a study in Japan involving 10,390 patients [26]. The prevalence of EIMs in patients with IBD was estimated to be 25–50% in Western countries, and EIMs were more common in CD than in UC [27]. Prideaux et al. reported that 3.7–24% of patients with IBD in East Asia had EIMs, and the prevalence was higher in India [2]. Wei et al. reported that EIMs occurred in 1% of CD cases and in 4.5% of UC cases in their hospital-based study in Taiwan [5, 24]. Our study found that 7.9% of patients with IBD had EIMs, which was similar to that in previous Asian reports. Concerning the prevalence of CRCs in patients with UC, it has been reported as 3–5% in Western countries and 0.0–2.2% in Asia [2]. Only a few studies on CRCs associated with CD have been reported in the literature, which demonstrated an increased risk in patients with colonic-type CD but without significance [28]. Eaden et al. conducted a meta-analysis study excluding Asian countries and found that the cumulative rate was 2%, 8%, and 18% in the first, second, and third decades, respectively [29]. Our present study showed that the incidence of CRCs in patients with IBD was 2.1%, which is comparable to that reported in previous Asian studies. This low incidence of CRCs might be related to the relatively short follow-up duration in many Asian studies. Thereby, we intend to continue following up our patients.

Because Asian countries are an endemic area for HBV infection, they have a higher seroprevalence of hepatitis B surface antigen (HBsAg) than North America and Europe do [30]. The seroprevalence of HBsAg in patients with IBD varies worldwide. In a report from China, the seroprevalence of HBsAg was 5.29% in patients with CD and 5.68% in patients with UC [31]. This study concluded that there was a higher seroprevalence of HBsAg in patients with IBD than in healthy controls. The Taiwanese government implemented a universal HBV mass vaccination program for infants in 1984. Thus, the seroprevalence of HBsAg in the general population was reported to be 15–20% before the HBV vaccination implementation and 13.7% in 2007 (after HBV vaccination implementation) [32, 33]. In young adults receiving HBV vaccination in Taiwan, the seroprevalence of HBsAg was reported to be 0.9% in 2011 and 1.1% in 2012 [34, 35]. Our present study showed that the seroprevalence of HBsAg was 13.3% in 158 patients with IBD with available data (CD: 11.3%, UC: 15.4%), which was similar to that in the general population in Taiwan. Our patients had a mean age at diagnosis of 38.4 years, which was older than the HBV vaccination program. Being an endemic area of HBV infection, the prophylactic use of antiviral agents for patients with IBD with HBV infection is an important issue in Taiwan. Moreover, data on the seroprevalence of HCV infection in patients with IBD are limited and conflicting. Coban et al. reported that the seroprevalence of HCV infection in patients with IBD was 39.8% in CD cases and 41.6% in UC cases in China, 23.8% in CD cases and 35.2% in UC cases in South Korea, and lower in Europe [36]. We found no HCV infection in 158 patients with available data, compared with the previously reported incidence (3.28%) in the general population in Taiwan [37].

A variety of serologic markers that are relevant to the diagnosis and treatment of IBD are emerging, including ANCAs, anti-*Saccharomyces cerevisiae* antibodies, anti-chitobioside carbohydrate antibodies, anti-laminaribioside carbohydrate antibodies, anti-mannobioside carbohydrate antibodies, and anti-outer membrane porins [38]. ANCAs, including p-ANCA and c-ANCA, are autoantibodies against neutrophil granules and monocyte lysosomes. Serum p-ANCA was first reported to be associated with UC and has been applied as a diagnostic tool for detecting IBD. Prideaux et al. conducted a review study and found that the prevalence of p-ANCA in patients with IBD was 6–38% in CD cases and 41–73% in UC cases [39]. Some studies have claimed that p-ANCA in UC was less prevalent in Asia than in Western countries [40, 41]. To our knowledge thus far, there is no report on the ANCA prevalence in patients with IBD in Taiwan. In our present study, we identified the prevalence of p-ANCA as 22.2% in patients with UC, which was similar to that in a Korean report (22.1%) but was lower than that in a report from China (43.4%) [42, 43]. Moreover, we also identified that the prevalence of c-ANCA was 1.6% in patients with UC, which was lower than that in previous reports from Jeddah (3%), Japan (39.2%), and England (31%) [44–46].

Concerning the treatment regimens, 5-ASAs and corticosteroids are highly preferred worldwide for the treatment of IBD. In the present study, 5-ASAs were the preferred regimen for the treatment of IBD, especially UC, which was consistent to that reported in other studies [47–49]. Concerning the use of immunomodulators, our present study demonstrated that immunomodulators were more often prescribed in patients with CD than in those with UC, which is comparable to the findings of other reports [48, 49]. With respect to the use of biologic agents, 85.0% of our patients with CD and 42.7% of patients with UC received biologic treatments, which were much higher than those in previous Western and Asian reports (3–25% vs. 8–11% in CD cases and 1–2% vs. 9% in UC cases) [40, 48–50]. Although many studies have demonstrated the efficacy of biologic agents for IBD, patients in some countries have to pay for biologic agents [51]. In Taiwan, we have a National Health Insurance system covering >99% of the population, which partially pays for the use of biologic agents. Thus, patients with IBD can usually receive treatment with biologic agents after National Health Insurance verification in Taiwan. Therefore, our insurance system might be one of the reasons for the higher use of biologic agents. The bowel resection rates in patients with IBD vary among different regions and countries [52, 53]. However, we found that the bowel resection rate in patients with IBD has decreased in some newer cohort studies. This might be attributed to medical advances, such as biologic agents and medical care, for patients with IBD. The bowel resection rate in our patients with CD was 38.8%, which was similar to that reported in a Korean study (35.5%) but was lower than that in European (38–61%) and Japanese (55%) reports after a 10-year follow-up [54–56]. Sato et al. reported that ileal-type CD had a significantly higher cumulative rate of surgery than the other types [55]. In our present study, 57.5% of patients with CD had the ileal type with poor disease behavior (stricturing and penetrating types were found in >80% of the patients), which may be the reason for the higher bowel resection rate despite more advanced medical care such as the use of immunomodulators and biologic agents. We also found a colectomy rate of 3.6% in patients with UC, which was lower than that reported in Korea (7.5%), Japan (14.5%), and Europe (2.8–24%) [50, 56, 57]. AVN of the hips is associated with significant morbidity, potentially causing severe pain and debilitation. In the literature, there were large variations in the incidence of hip AVN in patients with IBD. Corticosteroids are a known risk factor for hip AVN [58]. Freeman and Freeman reported that the incidence rate of hip AVN in all patients with CD was less than 0.5%, but it was 1% in male patients [59]. Recently, Rolston et al. reported that the incidence of hip AVN in patients with IBD was 2.1% [60]. They concluded that patients with IBD had a higher prevalence of AVN than patients without IBD. In our study, we reported a prevalence rate of hip AVN of 3.2% in all patients with IBD (3 CD cases and 3 UC cases), which was higher than that in previous reports. Our higher prevalence rate of hip AVN in patients with IBD may be due to the overuse and long-duration use of corticosteroids in Taiwan. Therefore, we suggest early tapering of corticosteroids and close surveillance for hip AVN in patients with IBD, especially those with prolonged use of corticosteroids.

## 5. Conclusions

In recent decades, the prevalence and incidence of IBD have been increasing but remain low in Taiwan. To date, only a few hospital-based studies have described the clinical characteristics and treatment outcomes of IBD in Taiwan. We conducted a retrospective hospital-based study in central Taiwan. We found that patients with IBD had a male predominance, lack of familial clustering, and lower prevalence of p-ANCA, EIMs, HCV infection, and CRCs. However, we observed a higher seroprevalence of HBsAg in patients with IBD than that reported in other Asian and Western countries. Thus, early detection of HBV infection and earlier antiviral prophylaxis for IBD are important issues in Taiwan. Moreover, our patients showed greater severity and higher rates of complications, use of biologic agents, hip AVN, and bowel resection than patients in other Asian and Western studies. Thus, we recommend early diagnosis and evaluation of complications, early use of immunomodulators and biologic agents, and avoidance of long-term corticosteroid use in patients with IBD. These precautions can help guide the management strategies to change the clinical course of patients with IBD in Taiwan. The limitations of our present study are its small sample size and retrospective nature. In the future, more prospective population-based studies at the national level are needed to identify the clinical characteristics contributing to the IBD prevalence and disease treatment outcomes in Taiwan.

## Figures and Tables

**Table 1:** Clinical characteristics of patients with inflammatory bowel disease.

Characteristics	Total IBD (*N* = 190)	CD (*n* = 80)	UC (*n* = 110)	*p* value
Male sex, *n* (%)	135 (71.1)	60 (75.0)	75 (68.2)	0.306
Mean age at diagnosis (years) (±SD)	38.4 ± 15.9	36.0 ± 17.4	40.1 ± 14.6	0.080
Cigarette smoking				0.002
Nonsmoking, *n* (%)	132 (69.5)	50 (62.5)	82 (74.5)	
Ever smoking, *n* (%)	32 (16.8)	11 (13.8)	21 (19.1)	
Current smoking, *n* (%)	26 (13.7)	19 (23.7)	7 (6.4)	
Alcohol consumption				0.194
Nondrinking, *n* (%)	156 (82.1)	70 (87.5)	86 (78.2)	
Ever drinking, *n* (%)	10 (5.3)	3 (3.8)	7 (6.4)	
Current drinking, *n* (%)	24 (12.6)	7 (8.7)	17 (15.4)	
Family history of IBD, *n* (%)	8 (4.2)	2 (2.5)	6 (5.5)	0.317
Extraintestinal manifestations, *n* (%)	15 (7.9)	7 (8.8)	8 (7.3)	0.709
Colorectal cancers, *n* (%)	4 (2.1)	1 (1.2)	3 (2.7)	0.484
Fistula formation, *n* (%)		22 (27.5)		
Positivity of HBsAg^^^, *n* (%)	21 (13.3)	9 (11.3)	12 (15.4)	0.444
Positivity of anti-HCV Ab^^^, *n* (%)	0 (0)	0 (0)	0 (0)	
Positivity of p-ANCA^∗^, *n* (%)			14 (22.2)	
Positivity of c-ANCA^∗^, *n* (%)			1 (1.6)	

Abbreviations: anti-HCV Ab: anti-hepatitis C virus antibody; c-ANCA: cytoplasmic anti-neutrophil cytoplasmic antibody; CD: Crohn's disease; HBsAg: hepatitis B surface antigen; IBD: inflammatory bowel disease; p-ANCA: perinuclear anti-neutrophil cytoplasmic antibody; UC: ulcerative colitis. ^^^There were 158 patients after excluding those with missing data. ^∗^There were 63 patients after excluding those with missing data.

**Table 2:** Montreal classification of patients with inflammatory bowel disease.

	CD (*n* = 80)	UC (*n* = 110)
UC disease extent		
E1: proctitis, *n* (%)		20 (18.2)
E2: left-sided colitis, *n* (%)		47 (42.7)
E3: extensive colitis, *n* (%)		43 (39.1)
CD disease phenotype		
L1: ileum, *n* (%)	46 (57.5)	
L2: colon, *n* (%)	6 (7.5)	
L3: ileo-colon, *n* (%)	27 (33.8)	
L4: UGI tract, *n* (%)	1 (1.3)	
CD disease behavior		
B1: nonstricturing, nonpenetrating^#^, *n* (%)	15 (18.8)	
B2: stricturing^#^, *n* (%)	48 (60.0)	
B3: penetrating^#^, *n* (%)	17 (21.3)	
P: perianal involvement, *n* (%)	17 (21.3)	

Abbreviations: CD: Crohn's disease; UC: ulcerative colitis; UGI: upper gastrointestinal. ^#^Includes cases with perianal involvement.

**Table 3:** Treatment regimens and clinical outcomes of patients with inflammatory bowel disease.

Treatment regimens and outcomes	Total IBD (*N* = 190)	CD (*n* = 80)	UC (*n* = 110)	*p* value
5-ASAs, *n* (%)	184 (96.8)	75 (93.8)	109 (99.1)	0.038
Corticosteroids, *n* (%)	132 (69.5)	64 (80.0)	68 (61.8)	0.007
Immunomodulators, *n* (%)	81 (42.6)	51 (63.8)	30 (27.3)	<0.001
Biologic agents, *n* (%)	115 (60.5)	68 (85.0)	47 (42.7)	<0.001
Bowel resection, *n* (%)	35 (18.4)	31 (38.8)	4 (3.6)	<0.001
Avascular necrosis of the hips, *n* (%)	6 (3.2)	3 (3.8)	3 (2.7)	0.484

Abbreviations: 5-ASAs: 5-aminosalicylic acids; CD: Crohn's disease; IBD: inflammatory bowel disease; UC: ulcerative colitis.

## Data Availability

The clinical data used to support the findings of this study are restricted by the institutional review board of the research ethics committee of China Medical University Hospital in order to protect the patients' privacy. Data are available from the corresponding author (Dr. Hsiang-Chun Lai, u9702451@cmu.edu.tw) for researchers who meet the criteria for access to confidential data.
